# Pannexin 1 Modulates Axonal Growth in Mouse Peripheral Nerves

**DOI:** 10.3389/fncel.2017.00365

**Published:** 2017-11-22

**Authors:** Steven M. Horton, Carlos Luna Lopez, Elisabeth Blevins, Holly Howarth, Jake Weisberg, Valery I. Shestopalov, Helen P. Makarenkova, Sameer B. Shah

**Affiliations:** ^1^Department of Orthopaedic Surgery, University of California, San Diego, La Jolla, CA, United States; ^2^Research Service, Veterans Affairs San Diego Healthcare System, San Diego, La Jolla, CA, United States; ^3^Department of Bioengineering, University of California, San Diego, La Jolla, CA, United States; ^4^Department of Ophthalmology, University of Miami, Miami, FL, United States; ^5^Department of Molecular Medicine, The Scripps Research Institute, La Jolla, CA, United States

**Keywords:** pannexin, purinergic signaling, dorsal root ganglion, axon, peripheral nerve

## Abstract

The pannexin family of channels consists of three members—pannexin-1 (Panx1), pannexin-2 (Panx2), and pannexin-3 (Panx3) that enable the exchange of metabolites and signaling molecules between intracellular and extracellular compartments. Pannexin-mediated release of intracellular ATP into the extracellular space has been tied to a number of cellular activities, primarily through the activity of type P2 purinergic receptors. Previous work indicates that the opening of Panx1 channels and activation of purinergic receptors by extracellular ATP may cause inflammation and apoptosis. In the CNS (central nervous system) and PNS (peripheral nervous system), coupled pannexin, and P2 functions have been linked to peripheral sensitization (pain) pathways. Purinergic pathways are also essential for other critical processes in the PNS, including myelination and neurite outgrowth. However, whether such pathways are pannexin-dependent remains to be determined. In this study, we use a Panx1 knockout mouse model and pharmacological inhibitors of the Panx1 and the ATP-mediated signaling pathway to fill gaps in our understanding of Panx1 localization in peripheral nerves, roles for Panx1 in axonal outgrowth and myelination, and neurite extension. Our data show that Panx1 is localized to axonal, myelin, and vascular compartments of the peripheral nerves. Knockout of Panx1 gene significantly increased axonal caliber *in vivo* and axonal growth rate in cultured dorsal root ganglia (DRG) neurons. Furthermore, genetic knockout of Panx1 or inhibition of components of purinergic signaling, by treatment with probenecid and apyrase, resulted in denser axonal outgrowth from cultured DRG explants compared to untreated wild-types. Our findings suggest that Panx1 regulates axonal growth in the peripheral nervous system.

## Introduction

The pannexin family of channels consists of three members—Panx1, 2, and 3—that enable the exchange of metabolites and signaling molecules between intracellular and extracellular compartments. There is a large body of evidence that Panx1 channels, together with extracellular adenosine triphosphate (ATP) and purinergic receptors, are involved in several physiological and pathological conditions(Iglesias et al., 2008; Dolmatova et al., 2012; Penuela et al., 2013; Adamson and Leitinger, 2014). Pannexin-mediated release of intracellular ATP into the extracellular space has been tied to a number of cellular activities, primarily through the activity of type P2 purinergic receptors, which elicit ATP-initiated ionotropic (P2X) or metabotropic (P2Y) cellular responses depending on the receptor subtype (Penuela et al., 2013; Makarenkova and Shestopalov, 2014). Among the diverse functions mediated downstream of pannexin/P2 signaling are calcium influx and depolarization in skeletal muscle (Cea et al., 2012), maintenance of vascular tone in arterial smooth muscle (Billaud et al., 2012), and the recruitment of leukocytes during inflammatory cascades (Makarenkova and Shestopalov, 2014; Lohman et al., 2015).

In the nervous system, Panx1 and 2 transcripts are found in the developing and mature central nervous system (CNS), including the brain, spinal cord, and eyes (Bruzzone et al., 2003; Dvoriantchikova et al., 2006; Raslan et al., 2016), and are implicated in neurogenesis, neural differentiation (Swayne et al., 2010; Wicki-Stordeur et al., 2012; Swayne and Bennett, 2016), and activation of inflammasomes (Silverman et al., 2009). In the peripheral nervous system (PNS), pannexins have primarily been studied in dorsal root (sensory) ganglia (DRG), in the context of neuropathic pain. Panx1 is expressed in sensory ganglia during all stages of development, including adults (Retamal et al., 2014; Raslan et al., 2016). Its expression in DRG increases following nerve injury (Zhang et al., 2015), a finding consistent with a reduction in injury-induced mechanical hypersensitivity following genetic knockout or chemical reduction of Panx1 expression (Zhang et al., 2015; Weaver et al., 2017). Several cell types, including neurons, satellite glial cells, and hematopoietic cells of the DRG are believed to contribute to pannexin-mediated peripheral pain pathways (Huang et al., 2013; Retamal et al., 2014; Zhang et al., 2015; Weaver et al., 2017), and there is significant cross-talk among these cells, as demonstrated by interactions between glial cells and neuronal cell bodies within sensory and other ganglia (Huang et al., 2013; Shibukawa et al., 2015; Spray and Hanani, 2017). Pannexin participation is hypothesized to occur via coupling with P2 receptor activity (Huang et al., 2013; Zhang et al., 2015), including P2X7 and P2X4 (Hung et al., 2012; Kawano et al., 2012), based on evidence for pannexin/P2 interactions in central sensitization pathways (Fabbretti, 2013; Bravo et al., 2014, 2015) and in sensory signaling pathways within sensory ganglia (Huang et al., 2013; Spray and Hanani, 2017).

Purinergic signaling has been reported to be essential for other critical processes in the PNS, including myelination of peripheral neurons (Faroni et al., 2014; Ino et al., 2015) and neurite outgrowth (Arthur et al., 2005; Vrbova et al., 2009). However, the role of Panx1 in axonal growth has not been tested. In this study, a Panx1 knockout mouse model and pharmacological inhibitors of ATP-mediated signaling were used to investigate roles for Panx1 in the PNS. Panx1 protein expression was found in axons, myelin, and the blood vessels of peripheral nerves. We found that axonal caliber in sciatic nerves and axonal density and growth rates in cultured DRG explants were increased in Panx1 knockout mice compared to wild-type controls. In addition, perturbation of pannexin mediated signaling by probenecid (pannexin inhibitor) or apyrase (that hydrolyzes ATP and ADP) treatment resulted in increased axonal density in cultured DRG explants compared to untreated explants. Our findings suggest that pannexin signaling (in particular Panx1) plays an important role in regulating neuronal morphology and neurite outgrowth in the PNS.

## Materials and methods

### Animal usage and primary tissue culture

All experiments were performed in accordance with the NIH Statement for the Use of Animals and were approved by the University of California, San Diego and The Scripps Research Institute Animal Care and Use Committees. Tissue was harvested from 10 to 14 week old wild-type (WT) and Panx1^−/−^ C57/Bl6 mice immediately following euthanasia by CO_2_. Male and female mice were pooled in equal numbers, to ensure that results were not skewed by gender-related factors. Panx1^−/−^ mice are functional knockouts that express a truncated form of active Panx1 subunits (Dvoriantchikova et al., 2012).

Segments of sciatic nerves between the sciatic notch and distal trifurcation were excised within 10 min of sacrifice. Harvested tissue was fixed by submersion in 10% formalin for 24 h at room temperature, followed by 15 and 30% sucrose in PBS for 24 h at 4°C. Following fixation, harvested tissue samples were frozen 2-Methylbutane (isopentane) (78-78-4, Sigma-Aldrich) cooled by liquid nitrogen, and stored at −80°C. Ten to twenty micrometers thick slices were sectioned using a cryostat (−20°C), mounted onto glass slides, and stored at −20°C.

Primary cell cultures of the thoracic and lumbar DRG were dissected out within 20 min of mouse sacrifice. Explants were seeded on 65 mm plastic tissue culture plates. One microliter Matrigel (356234, BD Bioscences) was used as a local adhesive beneath each explant. Explants were allowed to grow for 11 days in growth media consisting of 5% B-27 Supplement (17504-044, Gibco), 1% 200 mM 100X L-glutamine solution (SH30034, Hyclone), and 1% pen/strep/anti-mycotic (15240-062, Gibco) in Neurobasal-A (10888-022, Gibco) (modified from Liu et al., 2013; Bober et al., 2015). 0.05% nerve growth factor (NGF-7S, N0513, Sigma-Aldrich) was included in the growth media unless otherwise stated. To further investigate purinergic signaling pathways, WT DRG were also treated with media containing 2.5 units/ml apyrase (from potatoes, A7646, Sigma-Aldrich) or 50 μM probenecid (20061, Bioquest), 1 mg/mL ^10^Panx peptide, or 1 μM ATP for 7 days (Zhang et al., 2007; Kim et al., 2012; Petitjean et al., 2014). For all drug treatment groups, contralateral control explants were harvested and seeded in normal media, to confirm successful tissue culture for a given round of dissections.

### Immunolabeling

Prior to antibody labeling, slides or cultured explants were immersed in 10% formalin for 10 min, permeabilized in 0.2% Triton-X100 in PBS for 10 min, and blocked in 3% BSA and 10% fetal goat serum in PBS at room temperature for 30 min. Samples were then incubated overnight in 100 μg/mL of AffiniPure Fab IGG goat-anti-mouse antibody fragment (115-007-003, Jackson ImmunoResearch) in PBS at 4°C overnight, to reduce non-specific binding of murine-derived antibodies on mouse tissue samples. Samples were then incubated in primary antibodies overnight at 4°C, and incubated in appropriate secondary antibodies for 1 h at room temperature. 3x rinses with 0.05% Tween-20 in PBS at room temperature took place in between each step. Vectashield (H-1000, Vector Labs) was applied to each sample prior to placing coverslip. Primary antibodies used were: anti-β-tubulin isotype III mouse monoclonal antibody (T5076, Sigma-Aldrich: 1:500 dilution); anti-laminin rabbit polyclonal antibody (L9393, Sigma-Aldrich: 1:500 dilution); anti-myelin basic protein (MBP) rabbit polyclonal antibody (PA1-18324, Sigma-Aldrich: 1:200 dilution); anti-Panx1 rabbit polyclonal antibody (HPA016930, Sigma-Aldrich: 1:250 dilution). AlexaFluor 488 and AlexaFluor 594 goat-anti-mouse or goat-anti-rabbit secondary antibodies (Life Technologies, 1:200 dilution) specific for a given primary antibody were used to enable fluorescence imaging. All antibodies were diluted in blocking buffer. Negative controls with no antibody, primary antibody alone, or secondary antibody alone were used to confirm the specificity of labeling with each antibody.

### Microscopy

All stained samples including sectioned tissue on slides and cell culture dishes were imaged using a Leica DMI 6000 B confocal microscope. DRG explant cultures were imaged with a 20X/0.6NA objective lens. Sciatic nerve cross-section samples were imaged using a 63X/1.4NA objective lens with glycerol for fluid immersion. Line-averages of five scans were used to reduce background noise, and optimal slices for analysis were selected from z-stacks comprising 25 images captured at a spacing of 1 μm. Gain was kept constant to compare fluorescence intensity across genotypes.

Brightfield images of DRG explant cultures were imaged with a 20x/0.4 NA objective lens using a BZ-X700 Microscope (Keyence Corporation, of America, Itasca, IL). In order to measure axonal density and length; images were taken for samples at day 7 for a total of three explants per condition from at least two different animals (wild-type, knockout, wild-type + apyrase, wild-type + probenecid).

### Image processing

Co-localization between Panx1 and β3-tubulin in tissue sections was performed using ImageJ (National Institutes of Health; Just Another Co-localization Plug-in). Manders' coefficients M1 and M2 represented the overlap of Panx1 with β3-tubulin, and β3-tubulin with Panx1, respectively. The cross sectional area of axons was measured by binary thresholding of β3-tubulin labeling in a randomly selected 63x field in a given tissue section. All axons within this field were selected manually, and the area calculated from the number of connected pixels within this selection (ImageJ). At least 87 axons from three different animals were measured per genotype.

Growth rates were compared between WT and Panx1^−/−^ explants over an 11-day period. Bright-field images were taken of the explants and their projections every other day, and the lengths of the 10 longest axons per sample were measured using ImageJ. The number of projecting axons was counted and recorded for any samples with less than 10 axons. Axons from nine WT explants from three different mice and eight Panx1^−/−^ explants from three different mice were used for growth analysis. Branching was also compared between WT and Panx1^−/−^ mice, by analyzing images immunolabeled with anti-β3-tubulin antibody at the 11-day time point. Two circumferential lines were drawn at a radius 100 microns apart around the explant mass. Branching within this distance was measured as the ratio in the number of axonal segments crossing the inner and outer lines, with a higher ratio implying increased branching resulting in a higher ratio. The density of axons in wild-type, Panx1^−/−^, and probenecid, apyrase and ^10^Panx blocking peptide treated DRG explants was measured at the 7-day time point by binary thresholding (Otsu's method, using ImageJ) of bright field images in an area of ~200 × 200 pixels adjacent to each explant body, and calculating the area fraction of axon positive pixels. Explant masses were not included in quantification.

### Statistics

Means and standard errors were calculated for each measured parameter. Axonal lengths were compared between genotypes using a two-tailed heteroscedastic *t*-test. Axonal densites were compared among groups using a one-way ANOVA, with Tukey's HSD for *post-hoc* comparison, accounting for multiple comparisons. Axonal cross-sectional areas were compared between genotypes using the Welch's *t*-test, for non-Gaussian distributions. Significant differences between groups were indicated by *p*-values < 0.05.

## Results

### Panx1 protein expression in peripheral nerves

**Figure 1:**
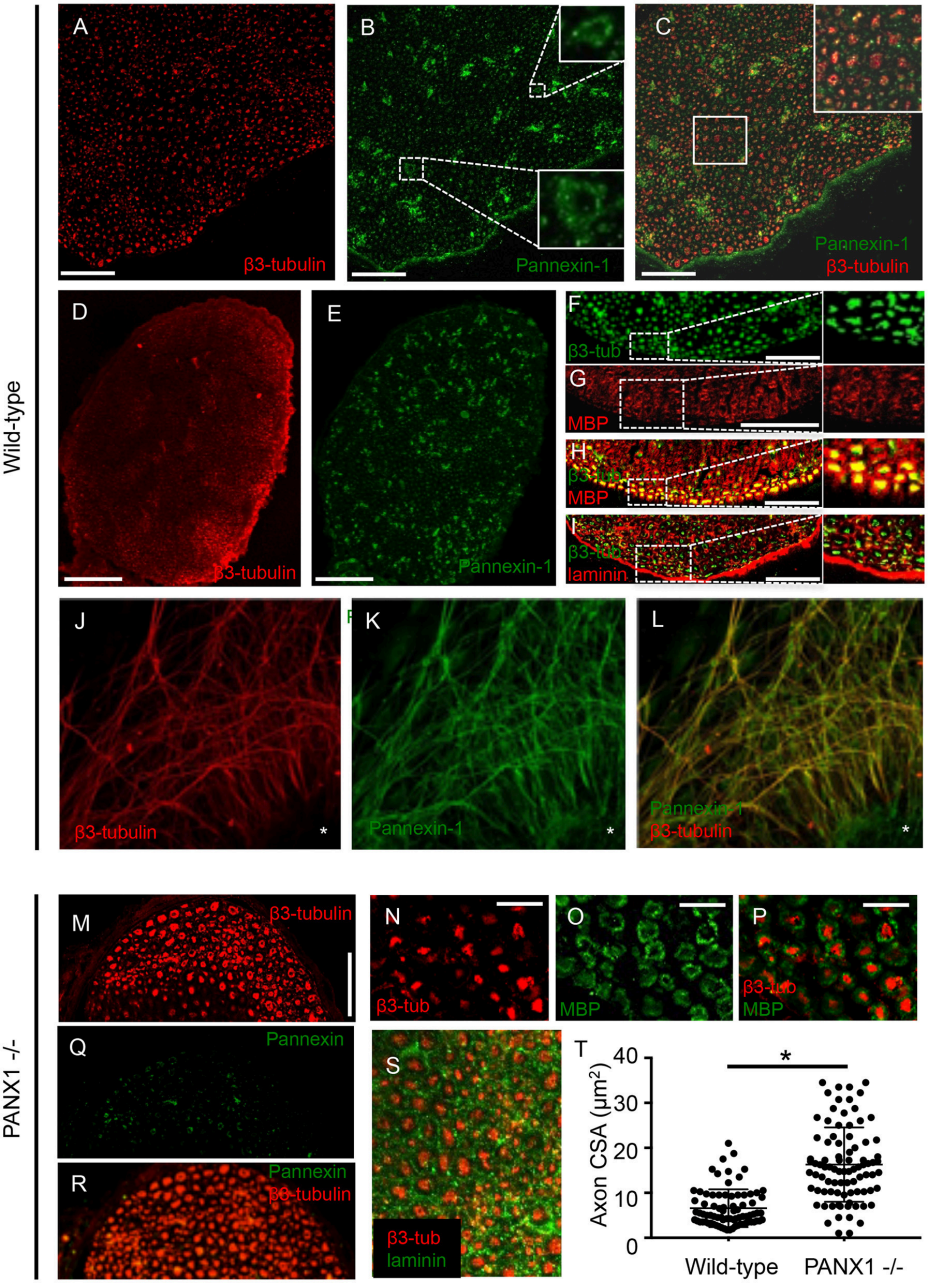
Panx1 is expressed in axons. **(A–I)** Immunolabeled sections from wild-type sciatic nerves. **(A–C)** β3-tubulin **(A)** and Panx1 **(B)** colocalize **(C)**. Insets in **(B,C)** show, respectively, enlargements of blood vessels and co-localizing Panx1 and β3-tubulin. **(D–E)** β3-tubulin **(D)** and Panx1 **(E)** colocalize over the entire cross-section of sciatic nerves, suggesting Panx1 localization to motor and sensory axons. **(F–H)** Serial sections show that β3-tubulin **(F)** and myelin basic protein (MBP) **(G)** colocalize **(H)**. Nerve fibers are separated by laminin-delineated sheaths **(I)**. **(J)** β3-tubulin immuno-labeling shows a characteristic punctate pattern within the nerve. β3-tubulin and Panx1 **(K)** co-localize **(L)** in cultured DRG explants from wild-type mice. Explant mass is indicated by an asterisk. **(M)** β3-tubulin labeling in Panx1 knockout nerves shows a characteristic punctate pattern, though axons appear to be larger. **(N–Q)** Axonal localization appears normal relative to myelin **(N–P)** and laminin **(Q). (R–S)** Panx1 labeling of Panx1 knockout nerves shows diffuse, non-specific labeling consistent with the truncated protein that results in functional knockout. **(T)** Quantification reveals that cross-sectional area is larger in PANx1^−/−^ axons than wild-type. ^*^Indicates significant difference. Bars: **(A–C)**, 50 μm; **(D–E)**, 100 μm; **(F–I)**, 50 μm; **(J–L)**, 100 μm; **(M)**, 50 μm; **(N–P)**, 25 μm.

To detect the localization of Panx1 in peripheral nerves, we first immuno-labeled cross-sections of wild-type mouse sciatic nerves. Panx1 labeling revealed a punctate pattern typical of axons in cross-section, which localized within or just beyond the boundaries of the neuron-specific β3-tubulin label (Figures 1A–C). Panx1 also localized to blood vessels, which were identified based on morphology (Figure 1B, insets). In addition, patches of Panx1 positive labeling were observed in the endoneurial (i.e., non-fiber) space (Figure 1C); the identity of cells to which these patches localize is unknown. Observed labeling across the entire cross-section of the sciatic nerve suggests Panx1 localization to both motor and sensory axons (Figures 1E,F). Quantitative analysis confirmed strong co-localization of Panx1 and β3-tubulin, with Mander's coefficients M1 (overlap of Panx1 positive pixels with β3-tubulin positive pixels) = 0.71 ± 0.08 and M2 (overlap of β3-tubulin positive pixels with Panx1 positive pixels) = 0.93 ± 0.02 (*n* = 3 nerves from 3 animals). While Panx1 labeling did not appear to span the entire thickness of the myelin sheath, in many fibers, Panx1 was localized to the peri-axonal region of myelin. Immuno-labeling of serial sections with antibodies against the myelin and laminin indicated typical neuronal fiber localization in wild-type nerves (Figures 1F–I). To confirm the axonal localization of Panx1, we also double-labeled projections in cultured DRG explants, which are strictly axonal, with antibodies against Panx1 and β3-tubulin (Figures 1J–L). Again, Panx1 and the axonal marker were strongly co-localized (M1 = 0.96 ± 0.01 and M2 = 0.85 ± 0.03; 9 explants from three animals). Panx1 also localized to structures within the explant mass, which contains neuronal cell bodies and assorted non-neuronal cells.

### Effects of Panx1 knockout on axonal morphology and outgrowth

Given the axonal localization of Panx1 and the importance of purigenic signaling pathways in myelination, we next assessed the morphology of sciatic nerves in Panx1^−/−^ mice using immunofluorescence. β3-tubulin labeling in Panx1^−/−^ nerve cross-sections revealed a characteristic punctate axonal pattern (Figure 1M). Axonal localization relative to myelin and laminin also appeared normal (Figures 1N–Q). Panx1 labeling in Panx^−/−^ nerves was diffuse, consistent with the mislocalization of the truncated form of Panx1 that results in the functional knockout (Figure 1R; Dvoriantchikova et al., 2012). Quantification of axonal caliber revealed a significant increase in axonal cross-sectional area in Panx1^−/−^ mice compared to wild-type mice (Figure 1S).

**Figure 2:**
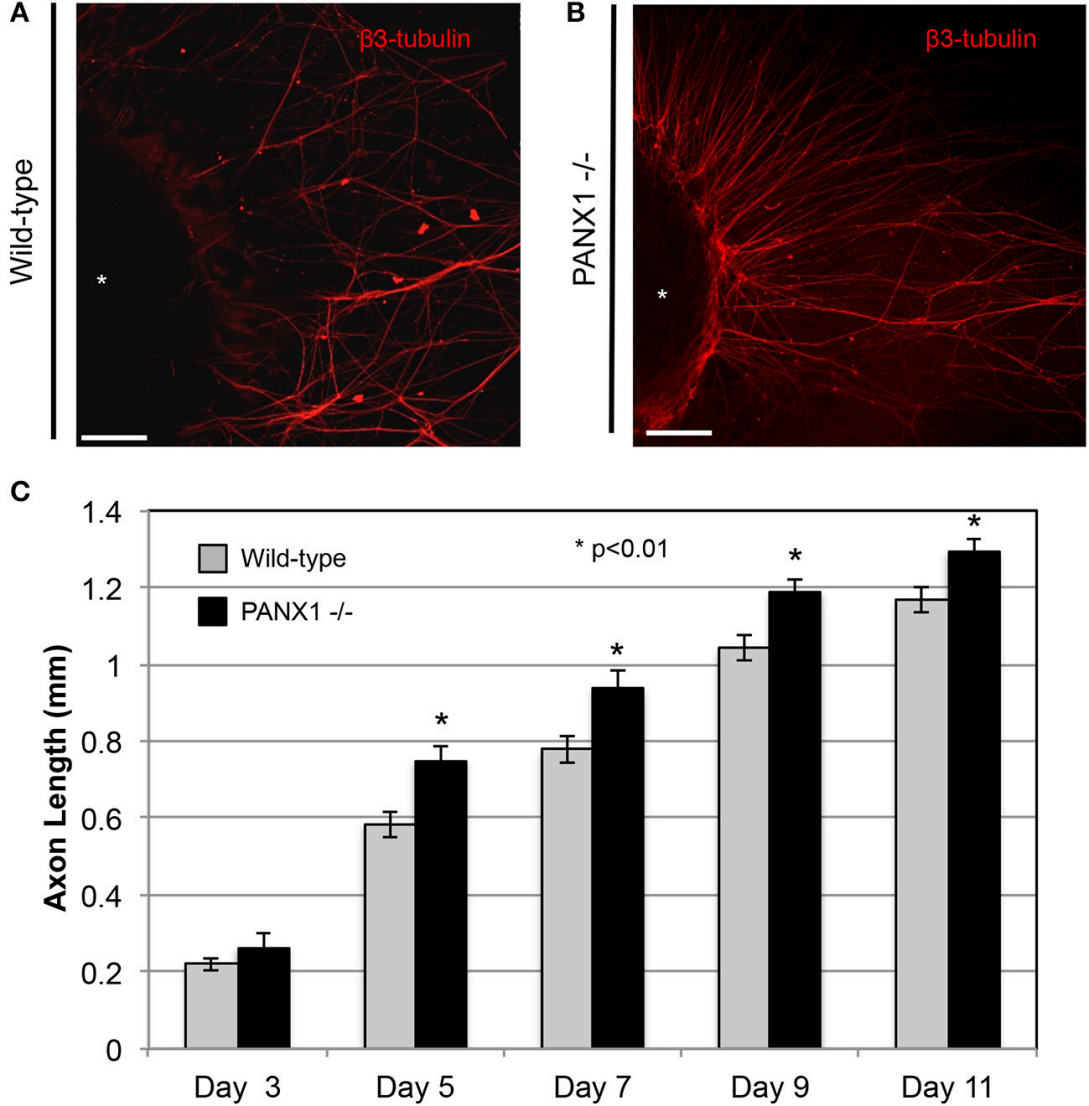
Axonal outgrowth from Panx1^−/−^ DRG explants is accelerated compared to that from wild-type explants. **(A)** Wild-type: Axons are shown in a fluorescence (β3-tubulin) image for Day 11. **(B)** Panx1^−/−^: Axons are shown in a fluorescence (β3-tubulin) image for Day 11. Bars: 50 μm. **(C)** Quantification of growth rates for axons of each genotype. Significance was assessed at each time point using Welch's *t*-test. ^*^P < 0.01.

As Panx1 knockout did not appear to adversely affect the structure of nerves *in situ* (i.e., in a normal developmental context), we then examined whether Panx1 was essential for regenerative outgrowth by comparing axonal extension in wild-type and Panx1^−/−^ DRG explants. Axon lengths of Panx1^−/−^ explants were significantly longer than those in wild-type explants at days 5, 7, 9, and 11 after DRG excision (Figures 2A–C; WT: *n* = 9 explants from three mice; Panx1^−/−^: *n* = 8 explants from three mice. Though we did not observe a statistically significant difference in axonal length at day 3, wild-type ganglia only had 5.4 ± 1.2 measurable axons per explant, while Panx1^−/−^ ganglia had 5.9 ± 1.3 axons per explant. Thus, Panx1^−/−^ axons may have experienced slightly accelerated initiation of axonal extension compared to wild-type axons. Branching analysis revealed a strong trend (*p* < 0.06) toward increased axonal branching in Panx1^−/−^ explants compared to wild-type explants at the 11 day time point, despite relatively low statistical power [branching ratios = 1.74 ± 0.16 (WT) vs. 2.34 ± 0.22 (Panx1^−/−^), *n* = 5 explants from three mice per group]. Thus, overall outgrowth appeared more robust in the absence of functional Panx1.

### Effects of pharmacological perturbation of purinergic signaling on axonal outgrowth

**Figure 3:**
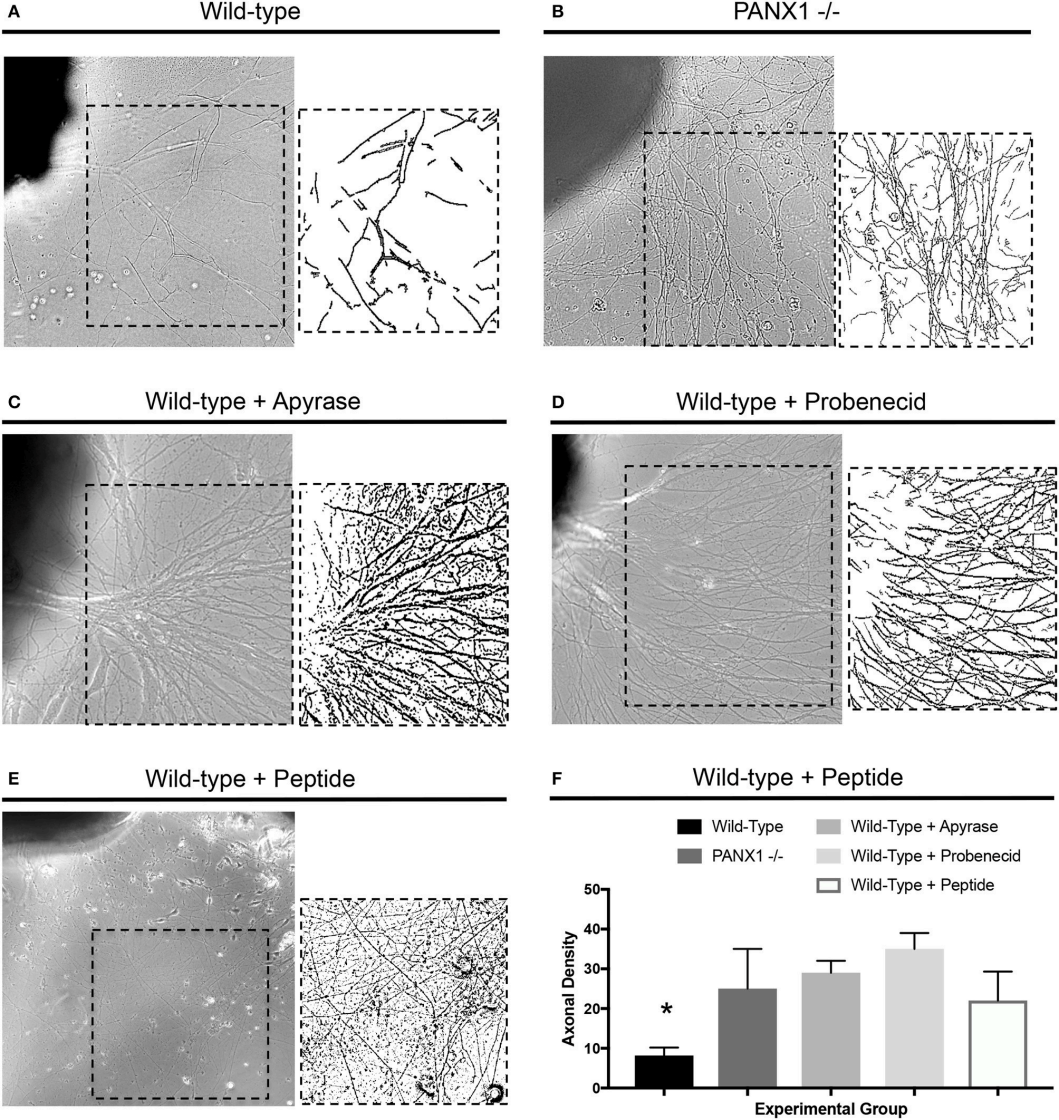
Disruption of purinergic signaling increases axonal density. **(A–D)** Samples of axonal outgrowth from **(A)** untreated wild-type, **(B)** untreated Panx1 knockout, **(C)** apyrase-treated wild-type, **(D)** probenecid-treated wild-type, and **(E)** ^10^Panx peptide-treated wild-type DRG explants. Insets show binarized images used to calculate axonal density. **(F)** Quantification of axonal density shows significant increases (*) in density in Panx1 knockout and apyrase, probenecid, and ^10^Panx-peptide-treated explants. Bars in brightfield images: 100 μm.

In light of the accelerated outgrowth of Panx1^−/−^ axons compared to wild-type axons, we next examined whether pharmacological perturbation of purinergic signaling would have similar effects on axonal growth. Treatment of wild-type DRG explants with modest levels of apyrase, which suppresses the activity of extracellular ATP, probenecid, reported to be an inhibitor of either pannexin, P2 receptor, or vanilloid channel activity (see Discussion), and ^10^Panx peptide, a specific inhibitor of pannexin function, all resulted in similar axonal densities as in Panx1^−/−^ explants, and dramatically and significantly higher axonal densities than in untreated explants (Figures 3A–F). In fact, these high densities prevented accurate measurement of axonal lengths in several samples, and so this parameter is not reported. In contrast, addition of 1 μM ATP to the culture media suppressed axonal outgrowth, especially in Panx1^−/−^ cultures (Supplementary Figures 1A,B).

To address the possibility that either NGF-mediated signaling or contact guidance through Matrigel may have over-ridden any inhibitory effect of Panx1 on axonal outgrowth, as controls, we cultured explants in the absence of NGF or within a 3D collagen substrate. These experimental systems have important methodological and technical features that preclude direct comparison with our Matrigel+NGF results; nevertheless, robust growth was observed in both genotypes in the absence of NGF and on collagen (Supplementary Figures 1C–F).

## Discussion

This study represents the first evaluation of the expression and localization of Panx1 protein in peripheral nerves, and assessment of the role of Panx1 on axonal morphology and outgrowth. We confirmed that Panx1 channels in peripheral nerves of adult mice are predominantly expressed in nerve fibers. Unexpectedly, despite previous evidence that Panx1 mediated purinergic signaling played an important role in myelination, Panx1 knockout did not adversely impact neuronal morphology; in fact, axonal caliber was increased in Panx1^−/−^ mice compared to wild-type mice (Figure 1). In addition, though purinergic pathways have been implicated in neurite outgrowth (Arthur et al., 2005; Vrbova et al., 2009), regenerative outgrowth in DRG explants was not only maintained, but accelerated in Panx1^−/−^ mice. We observed that Panx1 knockout, probenecid, ^10^Panx peptide, and apyrase treatment resulted in increased axonal density in cultured DRG explants, and ATP treatment resulted in suppressed axonal outgrowth. Thus, it appears that, in addition to modulating inflammatory and pain sensitization pathways in the DRG, Panx1 may also influence neuronal growth through purinergic signaling.

Our data confirmed the presence of Panx1 in the peripheral nervous system (Figure 1). Observed expression of Panx1 in axons of adult sciatic nerves and DRG explant cell bodies extended previous observations of Panx1 transcripts in neuronal cell bodies within the DRG. Observed Panx1 localization to larger blood vessels is consistent with its role in regulating vascular tone, via activity in smooth muscles (Billaud et al., 2012). In addition, patches of Panx1-positive immunolabeling external to axonal fibers (within the endoneurium) are an intriguing observation. These patches may reflect clusters of sensory axons (i.e., Remak bundles) that are scattered through the sciatic nerve, clusters of smaller blood vessels, fibroblasts, or resident progenitor cells—all of these cell populations are typical within nerves. Further studies will be required to identify such localization, sites of communication between neurons and myelinating cells within the axon, and the influence of these non-neuronal cells on axonal development and growth. From a methodological standpoint, the expression of Panx1 in non-neuronal structures may also provide a basis for the slight differences in Manders' coefficients calculated during co-localization analysis.

The presence of significantly larger, but otherwise normally organized axons in adult Panx1^−/−^ nerves suggests an important role for Panx1 in neuronal development. Regenerative growth does not fully recapitulate developmental pathways in neurons (Bosse et al., 2006; Harel and Strittmatter, 2006). Thus, we also explored the potential involvement of Panx1 channels in regenerative outgrowth of axons from DRG explants, which were axotomized upon excision prior to tissue culture. To our surprise, growth was more robust from Panx1^−/−^ explants, in terms of axonal extension, axonal branching, and axonal density. Importantly, we observed consistent outcomes with Panx1 knockout as well as pharmacological suppression of purinergic signaling elements, all of which also resulted in more robust axonal growth while application of ATP that activates purinergic receptors blocked this growth. It is still unclear whether probenecid is a specific inhibitor of pannexins (Silverman et al., 2008, 2009; Wicki-Stordeur and Swayne, 2013), or whether it can also inhibit the activity of P2 receptors in a pannexin-independent manner (Bhaskaracharya et al., 2014). Influences of enriched cell culture media on purinergic signaling are also not known. Nevertheless, the convergence of Panx1 knockout, probenecid, ^10^Panx peptide, and apyrase experiments provide substantial evidence for a regulatory role for purinergic signaling in axonal outgrowth.

Mechanisms of how impaired Panx1 activity could promote neuronal growth and branching remain to be elucidated; however, interactions between pannexins and the cytoskeleton (Boyce et al., 2014), and Arp2/3 in particular, have been documented (Wicki-Stordeur and Swayne, 2013), and Panx1 reduction has been reported to promote neurite outgrowth in neuronal cell lines and progenitor cells (Wicki-Stordeur and Swayne, 2013). In addition, pannexins have also been implicated in mechanical signaling pathways (Xia et al., 2012; Beckel et al., 2014; Krizaj et al., 2014). As axons undergo cyclic bouts of increased tension during extension, it is possible that reduced clearance of ATP from axons due to Panx1 knockout may perturb ATP-mediated cytoskeletal plasticity. Alternately, it was reported that upon reaching a threshold of extracellular ATP, Panx1 channels are endocytosed to down-regulate further ATP release and subsequent purinergic signaling (Boyce et al., 2015). It is possible that knocking-out Panx1 channels eliminates this negative feedback loop, leading to an increase in purinergic signaling. Such possibilities offer exciting avenues for future experiments. An additional topic of future testing is the relative contributions of Panx1 and pannexin-independent pathways for ATP release, including calcium homeostasis modulator (CALHM) ion channels (Cisneros-Mejorado et al., 2015; Ma et al., 2016) or ATP signaling through connexins, which play an important role in glial communication in sensory ganglia (Liu et al., 2014), and also in the auditory and visual nervous systems (Cowan et al., 2016; Hidaka, 2016; Akopian et al., 2017; Lukashkina et al., 2017).

We note that it is possible that Panx1 inactivation alone may not be sufficient to yield phenotypical or physiological changes, due to compensatory upregulation of Panx2 and 3 (Penuela et. al., 2012). However, this seems unlikely given the lack of data on expression of Panx2 or−3 in the adult PNS, as well as the predominantly cytoplasmic localization of Panx2 and substantial differences in the cellular localization between Panx1 and Panx2 (Boassa et al., 2014; Le Vasseur et al., 2014). Finally, it should be noted that connexins/pannexins play roles in other homeostatic functions, including neurotransmitter release and ion balance, which may also be reflected in our observations (Thompson and Macvicar, 2008; Bennett et al., 2012).

In summary, our study has revealed new high resolution data on the intracellular localization and homeostatic function of Panx1 in the peripheral nervous system. In particular, Panx1 and purinergic signaling appear to play an important role in modulating axonal geometry and outgrowth. Thus, additional study of pannexins in the PNS offers an interesting and exciting area of future research, including possible roles in peripheral nerve repair and regeneration.

## Author contributions

Experiments were designed by SMH, VIS, HPM, and SBS. Experiments were executed by SMH, CLL, EB, HH, and JW. Data was analyzed by SMH, CLL, EB, HH, JW, VIS, HPM, and SBS. Manuscript was written by SMH and SBS. Manuscript was edited and approved by all authors.

### Conflict of interest statement

The authors declare that the research was conducted in the absence of any commercial or financial relationships that could be construed as a potential conflict of interest.

## Supplementary material

Supplementary Figure 1 **(A,B)** Wild-type **(E)** and Panx1 knockout **(F)** explants grown in the presence of 1 μM ATP show a marked reduction in axonal outgrowth, especially in Panx1 knockout explants. **(C,D)** β3-tubulin labeled axons are seen in **(C)** Matrigel and **(D)** Collagen, indicating outgrowth within different substrates. Bars: 100 μm. **(E,F)** Wild-type **(E)** and Panx1 knockout **(F)** explants grown in NGF-free media show robust outgrowth. Bars: 500 μm.
