# Supplementary figures and images for: Pannexin 1 Modulates Axonal Growth in Mouse Peripheral Nerves
Source: Front Cell Neurosci. 2017 Nov 22;11:365. doi: 10.3389/fncel.2017.00365 (PMC5702652; doi:10.3389/fncel.2017.00365)

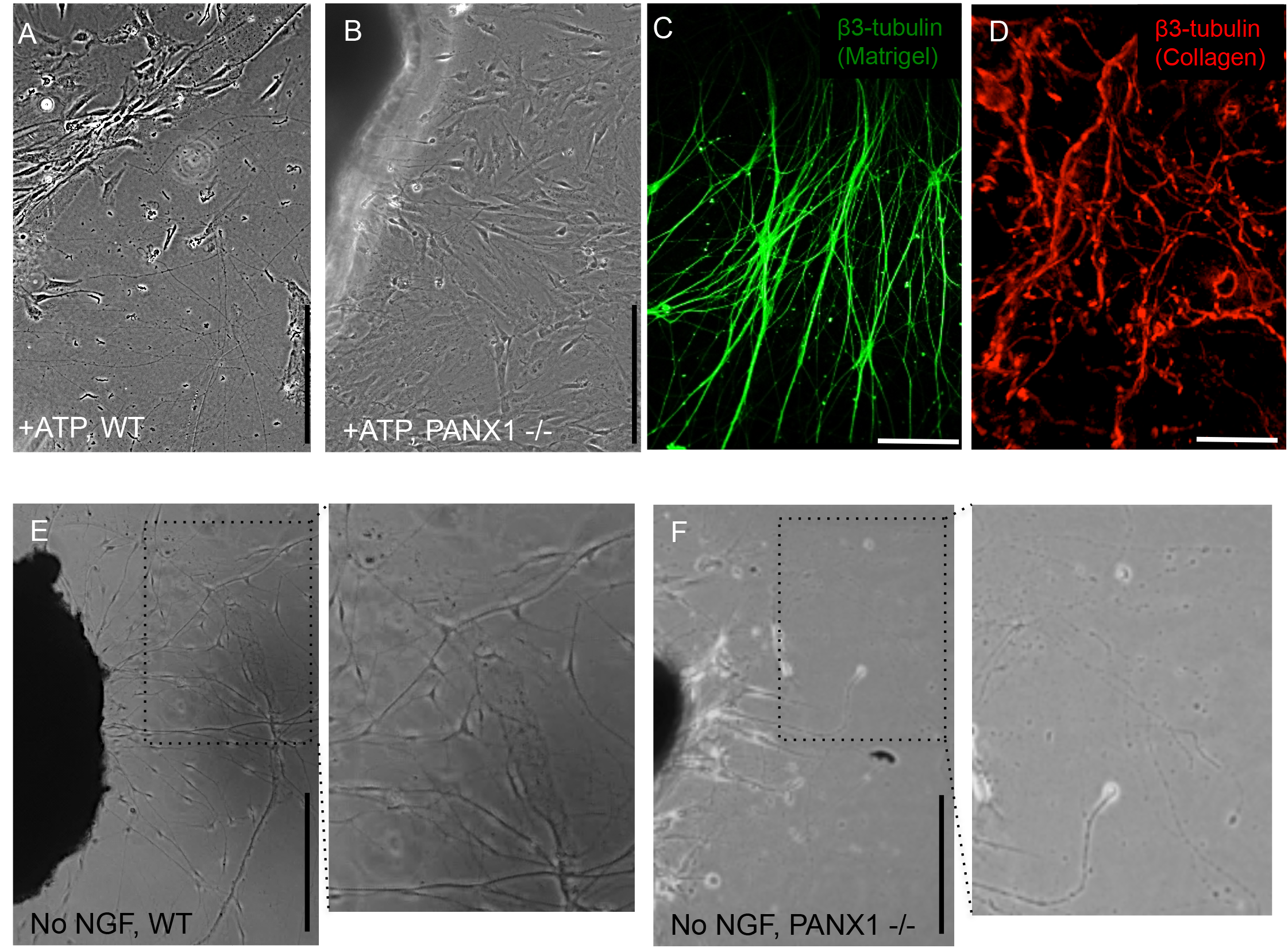

Supplement: Supplementary Figure 1 — (A,B) Wild-type (E) and Panx1 knockout (F) explants grown in the presence of 1 μM ATP show a marked reduction in axonal outgrowth, especially in Panx1 knockout explants. (C,D) β3-tubulin labeled axons are seen in (C) Matrigel and (D) Collagen, indicating outgrowth within different substrates. Bars: 100 μm. (E,F) Wild-type (E) and Panx1 knockout (F) explants grown in NGF-free media show robust outgrowth. Bars: 500 μm. [file Image1.tif]
